# Gemcitabine chemoresistance and molecular markers associated with gemcitabine transport and metabolism in human pancreatic cancer cells

**DOI:** 10.1038/sj.bjc.6603559

**Published:** 2007-01-16

**Authors:** Y Nakano, S Tanno, K Koizumi, T Nishikawa, K Nakamura, M Minoguchi, T Izawa, Y Mizukami, T Okumura, Y Kohgo

**Affiliations:** 1 Third Department of Internal Medicine, Asahikawa Medical College, Asahikawa, Japan; 2 Department of General Medicine, Asahikawa Medical College, Asahikawa, Japan

**Keywords:** pancreatic cancer, ribonucleotide reductase, deoxycytidine kinase, nucleoside transporter, gemcitabine resistance, predictive marker

## Abstract

To identify predictive molecular markers for gemcitabine resistance, we investigated changes in the expression of four genes associated with gemcitabine transport and metabolism during the development of acquired gemcitabine resistance of pancreatic cancer cell lines. The expression levels of human equilibrative nucleoside transporter-1 (*hENT1*), deoxycytidine kinase (*dCK*), *RRM1*, and *RRM2* mRNA were analysed by real-time light cycler-PCR in various subclones during the development of acquired resistance to gemcitabine. Real-time light cycler-PCR demonstrated that the expression levels of either *RRM1* or *RRM2* progressively increased during the development of gemcitabine resistance. Expression of *dCK* was slightly increased in cells resistant to lower concentrations of gemcitabine, but was decreased below the undetectable level in higher concentration-resistant subclones. Expression of *hENT1* was increased in the development of gemcitabine resistance. As acquired resistance to gemcitabine seems to correlate with the balance of these four factors, we calculated the ratio of *hENT1* × *dCK*/*RRM1* × *RRM2* gene expression in gemcitabine-resistant subclones. The ratio of gene expression decreased progressively with development of acquired resistance in gemcitabine-resistant subclones. Furthermore, the expression ratio significantly correlated with gemcitabine sensitivity in eight pancreatic cancer cell lines, whereas no single gene expression level correlated with the sensitivity. These results suggest that the sensitivity of pancreatic cancer cells to gemcitabine is determined by the ratio of four factors involved in gemcitabine transport and metabolism. The ratio of the four gene expression levels correlates with acquired gemcitabine-resistance in pancreatic cancer cells, and may be useful as a predictive marker for the efficacy of gemcitabine therapy in pancreatic cancer patients.

Pancreatic adenocarcinoma is a common cancer with extremely poor prognosis. In the past few years, gemcitabine, a novel pyrimidine nucleoside analogue, has become the standard chemotherapeutic agent used in patients with pancreatic cancer (Rothenberg *et al*, 1996; Burris *et al*, 1997). However, even with this drug, most pancreatic adenocarcinomas exhibit an inexorable disease progression, and the clinical impact of gemcitabine remains modest owing to a high degree of inherent and acquired chemoresistance (Carmichael *et al*, 1996; Li *et al*, 2004). No clinical molecular markers have previously been shown that can predict a benefit from gemcitabine chemotherapy, and patients are treated empirically until disease progression or worsening therapy performance. Therefore, it is extremely important to determine predictive molecular markers of gemcitabine resistance for more effective treatment of these tumours.

Gemcitabine has a complex pathway of metabolism, and there are many mechanisms that can contribute to gemcitabine cytotoxicity and/or chemoresistance (Habiro *et al*, 2004; Koizumi *et al*, 2005). In recent studies performed on human cancer cell lines, human equilibrative nucleoside transporter-1 (hENT1) was found to be the major gemcitabine transporter (Garcia-Manteiga *et al*, 2003). If gemcitabine is not transported into the cell via hENT1 it cannot inhibit cell growth (Mackey *et al*, 1998; Rauchwerger *et al*, 2000), but increased hENT1 abundance facilitates efficient cellular entry of gemcitabine and confers increased cytotoxicity (Mackey *et al*, 1999; Ritzel *et al*, 2001). Inside the cell, gemcitabine is phosphorylated by deoxycytidine kinase (dCK) in a rate-limiting step. Deficiency in dCK activity has been considered to be one of the main mechanisms responsible for the development of resistance to gemcitabine. Another factor in gemcitabine resistance is the overexpression of ribonucleotide reductase (RR). Ribonucleotide reductase is mainly responsible for the conversion of ribonucleosides to deoxyribonucleoside triphosphates (dNTPs), which are essential for DNA polymerisation and repair (Cory and Sato, 1983; Thelander and Berg, 1986; Fan *et al*, 1996; Zhou *et al*, 1998). RR consists of the dimerised large and small subunits, M1 and M2, respectively. The M1 subunit possesses a binding site for enzyme regulation (regulatory subunit), and the M2 subunit is involved with RR activity (catalytic subunit). Although it has been proposed that the genes for gemcitabine transport and metabolism are involved in the mechanism of cellular resistance to gemcitabine, it is not fully understood how gemcitabine influences its own transport and metabolism in the process of acquired resistance. Understanding alterations in expression of genes, which characterise the response of cancer cells to gemcitabine treatment in the process of acquired resistance, would allow us to improve therapeutic strategies for pancreatic cancer.

In this study, we established various gemcitabine-resistant subclones of human pancreatic cancer cell lines, and investigated changes in gene expression associated with gemcitabine transport and metabolism, that is, *hENT1*, *dCK*, *RRM1*, and *RRM2* mRNA, in the development of gemcitabine resistance. Quantitative RT–PCR analysis showed that the balance of the four gene expression levels is associated with inherent and acquired resistance to gemcitabine in pancreatic cancer cells. Resistance of cancer cells to gemcitabine is determined by the ratio of these gene expression levels, but not predicted by that of a single gene. The ratio of gene expression might be useful as a predictive marker for the efficacy of gemcitabine therapy in pancreatic cancer patients.

## MATERIALS AND METHODS

### Chemicals

Gemcitabine was a gift from Eli Lilly Pharmaceuticals (Indianapolis, IN, USA). All other chemicals were of analytical grade and commercially available.

### Cell culture and establishment of gemcitabine-resistant pancreatic cancer cells

Eight human pancreatic adenocarcinoma cell lines, PK1, PCI43, KLM1, PK8, PK9, MIAPaCa2, KP1N, and BxPC3, were used in this study. PK1, KLM1, PK8, and PK9 cell lines were obtained from the Cell Resource Center for Biochemical Research (Tohoku University, Sendai, Japan). KP1N and MIAPaCa2 cell lines were purchased from the Health Science Research Resources Bank (Osaka, Japan). The PCI43 cell line was provided by Dr H Ishikura at Hokkaido University (Sapporo, Japan). PK1, PCI43, KLM1, PK8, PK9, KP1N, and BxPC3 were grown in RPMI 1640 media (Gibco, Paisley, Scotland) supplemented with 10% heat-inactivated fetal bovine serum in a humidified 5% CO_2_ incubator at 37°C. The MIAPaCa2 cell line was cultured in Dulbecco's modified Eagles medium (DMEM). Gemcitabine-resistant cells were generated by exposing the PCI43, PK1, and KLM1 cell lines to incrementally increasing gemcitabine concentrations starting at 3 nM. As the cells adapted to the drug, the gemcitabine concentration was doubled. The intermediate resistant variants were cultured for at least 4 weeks. The cell lines were named as follows: G for gemcitabine, followed by the nM concentration at which the cell line grew logarithmically. The most resistant variants were PCI43-G4000, PK1-G4000, and KLM1-G4000 and were resistant to continuous exposure to gemcitabine at 4000 nM. Experiments were performed using cells in the exponential phase of growth.

### Drug cytotoxicity assay

The relative cytotoxicity of gemcitabine in each cell line was assessed with a WST-1 assay using a Cell Counting Kit (Dojindo Laboratories, Kumamoto, Japan). This assay is based on the reduction of a tetrazolium compound to a soluble derivative by the dehydrogenase enzymes of metabolically active cells. The absorbance (450 nm) is directly proportional to the number of living cells in culture. Cells were added to 96-well tissue culture plates (3 × 10^3^ cells/well) overnight and exposed to increasing concentrations (10^−2^ 10^3^ *μ*M) of gemcitabine for 72 h, after which the number of remaining living cells was determined according to the manufacturer's instructions. Chemosensitivity was expressed as the drug concentration that inhibited cell proliferation by 50% (IC_50_ values) and was determined from concentration–effect relationship.

### Quantitative LightCycler RT–PCR

Total RNA was extracted from each cell line and gemcitabine-resistant subclones using the RNeasy Protect Mini Kit (Qiagen, Hilden, Germany) with the RNase-Free DNase Set (Qiagen) according to the manufacturer's instructions. Complementary DNA (cDNA) was produced from 1 *μ*g of RNA using an Oligo (dT)_12−18_ Primer (Invitrogen, Carlsbad, CA, USA) and MMLV reverse transcriptase (Promega, Madison, WI, USA). Primers for *hENT1*, *dCK*, *RRM1*, *RRM2*, and *glyceraldehyde-3-phosphate dehydrogenase (GAPDH)* were based on the sequence of each gene (Entrez-PubMed) and designed by the program Primer 3. Oligonucleotides used as PCR primers are summarised in Table 1. Quantitative RT–PCR was performed in a LightCycler system (Roche Molecular Biochemicals, Mannheim, Germany) using SYBR Green fluorescence. In this system, all reactions were run in glass capillaries with a total volume of 20 *μ*l. The reaction mixture consisted of 2 *μ*l of FastStart DNA Master SYBR Green I, SYBR Green I dye, and 10mM MgCl_2_ (Roche Diagnostics GmbH, Mannheim, Germany). Primers were added to a final concentration of 3 4 *μ*M. In each experiment, 1 *μ*g of extracted RNA from the cells was reverse transcribed to generate cDNAs that were then diluted 1:10. Finally, 5 *μ*l of the diluted cDNA was added to a capillary tube. One positive control, one negative control, and standards were included in each run. The PCR programs started with a preincubation step for activation of the FastStart enzyme, then continued with amplification, and ended with melting curve analysis. The temperature transition rate was 0.1°C s^−1^. The preincubation and amplification programmes for each line were as follows: *hENT1*, preincubation at 95°C for 10 min and amplification with 40 cycles of 95°C for 10 s, 60°C for 10 s, and 72°C for 10 s; *dCK*, preincubation at 95°C for 10 min and amplification with 40 cycles of 95°C for 10 s, 56°C for 10 s, and 72°C for 17 s; *RRM1*, preincubation at 95°C for 10 min and amplification with 40 cycles of 95°C for 10 s, 58°C for 10 s, and 72°C for 10 s; *RRM2*, preincubation at 95°C for 10 min and amplification with 40 cycles of 95°C for 10 s, 60°C for 10 s, and 72°C for 6 s; *GAPDH*, preincubation at 95°C for 10 min and amplification with 40 cycles of 95°C for 10 s, 55°C for 10 s, and 72°C for 13 s. All programs were then followed by a heating step at 95°C and a cooling step at 65°C for 15 s each. Each experiment using quantitative RT–PCR was performed in triplicate. The expression of *hENT1*, *dCK*, *RRM1*, and *RRM2* mRNA was quantified relative to *GAPDH* expression.

The Roche software uses the second derivative maximum method to calculate the fractional cycle numbers where the fluorescence rises above background (crossing point, *C*_p_), that is, the point at which the rate of change of fluorescence is fastest. For the standard curve, CpS are plotted vs log concentration for the standards. This standard curve is used to estimate the concentration of each sample. The standard curves were saved in a coefficient file that was used by the relative quantification software from Roche to calculate the mRNA levels relative to *GAPDH*. This program also corrected for the differences in efficiency of the PCR reaction for each target.

### Statistical analysis

Data are expressed as means±s.d. Analysis was performed using the Mann–Whitney *U*-test (two-tailed) for nonparametric data. Correlations between nonparametric data were analysed by the Spearman correlation test.

## RESULTS

### Development of acquired gemcitabine resistance in pancreatic cancer cell lines, PCI43, PK1, and KLM1

Resistance to gemcitabine was successfully induced in the PCI43, PK1, and KLM1 cell lines via exposure to stepwise increases in gemcitabine concentration. Gemcitabine-resistant PCI43, PK1, and KLM1 cells were developed by continuous exposure to increasing concentrations of gemcitabine over a period of 6 months, starting with an initial concentration of 3 nM. The resistant cells obtained, PCI43-G4000, PK1-G4000, and KLM1-G4000, were viable in medium containing 4000 nM gemcitabine. Resistant cells showed no apparent morphologic differences or difference in growth rate compared with the parental cells.

The IC_50_ values and resistance ratios for parental cells and gemcitabine-resistant cells are listed in Table 2. The IC_50_ values of gemcitabine for the parental PCI43, PK1, and KLM1 cells were 350, 160, and 80 nM, respectively. PCI43-G4000, PK1-G4000, and KLM1-G4000 cells were 157-, 625-, and 2625-fold less sensitive to gemcitabine than the parental cell lines, respectively.

### Analysis of *hENT1*, *dCK*, *RRM1*, and *RRM2* mRNA expression by real-time light cycler-PCR in different gemcitabine-resistant cell lines

**Figure 1:**
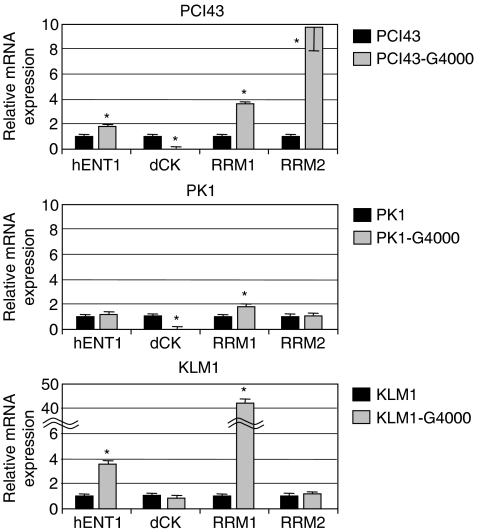
mRNA expression relative to *GAPDH* in parental and gemcitabine-resistant cells of PCI43, PK1, and KLM1 cell lines, respectively. Each IC_50_ value is the mean of the values in three independent sensitivity tests performed in quadruplicate. Expression levels are relative to expression of *GAPDH*. *P<0.05 to parental cells. Bars, s.d.

**Figure 2:**
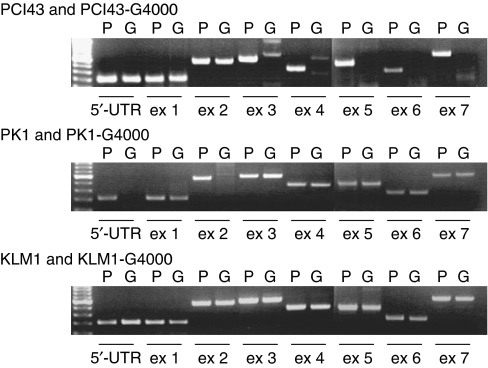
Analysis of dCK PCR product of parental cells and PCI43-G4000, PK1-G4000, and KLM1-G4000, on agarose gel electrophoresis stained with ethidium bromide. The 5′-untranslated region and complete coding sequence of exons 1–7 of the human *dCK* gene were amplified. P: parental cells, G: gemcitabine resistant.

To evaluate the expression of *hENT1*, *dCK*, *RRM1*, and *RRM2* mRNA, real-time light cycler-PCR was performed in a quantitative manner. The mRNA was extracted from each gemcitabine-resistant cell line and analysed via light cycler. Glyceraldehyde-3-phosphate dehydrogenase was used as an internal control. The quantitative data were summarised from the means of the data gathered from the three experiments (Figure 1). Real-time light cycler-PCR demonstrated that PCI43-G4000 cells had approximately 4- and 10-fold increases in the levels of *RRM1* and *RRM2* mRNA compared with the parental cells, respectively. PK1-G4000 cells had approximately a two-fold increase in levels of *RRM1* mRNA, but there was no increase of *RRM2* mRNA compared with parental cells. Similarly, KLM1-G4000 cells had approximately a 42-fold increase in levels of *RRM1* mRNA, but no increase of *RRM2* mRNA compared with parental cells. *Human equilibrative nucleoside transporter*-1 gene expression was significantly increased compared with parental cells in PCI43-G4000 and KLM1-G4000 cells, but not in PK1-G4000 cells. Expression of *dCK* mRNA was not detected by real-time light cycler-PCR in either PCI43-G4000 or PK1-G4000 cells, and no changes in *dCK* mRNA expression were observed in KLM1-G4000 cells. To determine the cellular modification responsible for *dCK* mRNA downregulation, seven exons of the *dCK* gene as well as the 5′-untranslated regions were amplified by PCR as described previously (Galmarini *et al*, 2004). In PCR products obtained using genomic DNA from cells, as shown in Figure 2, a partial deletion of the *dCK* gene was amplified in both PCI43-G4000 and PK1-G4000 cells. No deletion of the *dCK* gene was detected in parental cell lines or KLM1-G4000 cells.

### Gene expression changes in the process of acquired gemcitabine resistance by real-time light cycler-PCR in pancreatic cancer cells

**Figure 3:**
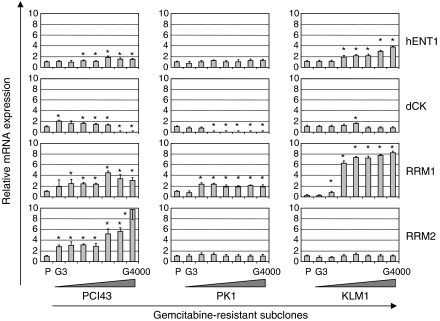
Relationship between gemcitabine resistance and expression levels of *hENT1*, *dCK*, *RRM1*, and *RRM2* mRNA in the process of acquired gemcitabine resistance in gemcitabine-resistant PCI43, PK1, and KLM1 subclones. Relative mRNA expression to *GAPDH* was calculated. The value of relative mRNA expression in parental cells was assigned as 1.0. Each point represents the mean±s.d. of quadruplicate determinations per plate repeated in triplicate. P: parental cells, G: gemcitabine-resistant. ^*^P<0.05 to parental cells. *Bars*, s.d.

**Figure 4:**
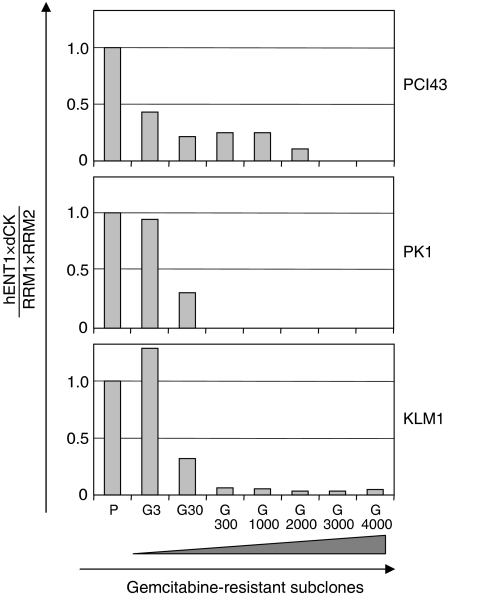
Changes in the *hENT1* × *dCK*/*RRM1* × *RRM2* expression ratio in gemcitabine-resistant PCI43, PK1, and KLM1 subclones. Value of the ratio in parental cells was set as 1.0. The ratio of *hENT1* × *dCK*/*RRM1* × *RRM2* expression progressively decreased in the process of acquired gemcitabine resistance. P: parental cells, G: gemcitabine resistant.

It is not fully understood how gemcitabine influences its own transport and metabolism in the development of gemcitabine resistance. To clarify the changes of gene expression in the process of acquired resistance, we established seven gemcitabine-resistant subclones of the PCI43, PK1, and KLM1 cell lines. Examination of the gemcitabine-resistant PCI43 subclones revealed a proportional increase in *RRM2* message with increasing gemcitabine exposure (Figure 3). *RRM1* mRNA showed a two- to four-fold up regulation of expression levels. Significant increases in *RRM1* and *RRM2* gene expression induced by gemcitabine were detected in the subclones PCI43-G30 and -G3, respectively. No *dCK* mRNA could be detected in subclones PCI43-G3000 and -G4000, which had higher resistance to gemcitabine, but the *dCK* gene was slightly increased at lower concentrations of gemcitabine. The *hENT1* gene was slightly increased in the latter phase of development of acquired resistance. In the gemcitabine-resistant PK1 subclones, a slight increase in *RRM1* mRNA was detected, but no increase in the *RRM2* gene was observed. No *dCK* mRNA could be detected in subclones PK1-G300 to -G4000. The gemcitabine-resistant KLM1 subclones showed marked upregulation in *RRM1* mRNA, but there was no increase in the *RRM2* gene. *dCK* mRNA could be detected in all KLM1 subclones. These results suggest that acquired resistance of pancreatic cancer cells to gemcitabine may be determined by the balance of these four factors, but is not predicted by that of a single gene. Decreased *hENT1* or *dCK* has been reported to promote gemcitabine resistance (Ruiz van Haperen *et al*, 1994; van der Wilt *et al*, 2000; Garcia-Manteiga *et al*, 2003; Jordheim *et al*, 2003; Galmarini *et al*, 2004). In contrast, increased expression of RR has been reported to be associated with gemcitabine resistance in human tumour cells (Goan *et al*, 1999; Jung *et al*, 2001). For these reasons, we calculated the ratio of *hENT1* × *dCK*/*RRM1* × *RRM2* gene expression in gemcitabine-resistant subclones. As shown in Figure 4, the ratio of *hENT1* × *dCK*/*RRM1* × *RRM2* expression progressively decreased in the process of acquired gemcitabine resistance.

### Correlation of the hENT1 × dCK/RRM1 × RRM2 ratio with gemcitabine chemosensitivity in pancreatic cancer cell lines

**Figure 5:**
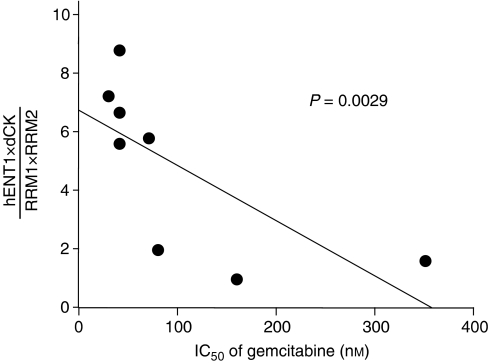
Significant correlation between the IC_50_ of gemcitabine and the value of *hENT1* × *dCK*/*RRM1* × *RRM2* expression ratio in eight pancreatic cancer cell lines (*P*=0.0029). Cells with a higher *hENT1* × *dCK*/*RRM1* × *RRM2* expression ratio showed higher gemcitabine chemosensitivity, whereas cells with a lower ratio showed higher chemoresistance.

To determine whether the *hENT1* × *dCK*/*RRM1* × *RRM2* ratio correlated with gemcitabine sensitivity, we examined the relative mRNA expression of *hENT1*, *dCK*, *RRM1*, and *RRM2* to *GAPDH* in eight human pancreatic cancer cell lines. Correlations of IC_50_ values and relative levels of gene expression in each cell lines are summarised in Table 3. We found that the IC_50_ values of gemcitabine did not significantly correlate with relative expression levels of *hENT1*, *dCK*, *RRM1*, or *RRM2*. Next, the ratio of *hENT1* × *dCK*/*RRM1* × *RRM2* expression was calculated and the correlation with gemcitabine sensitivity was determined (Figure 5). Cells with a higher *hENT1* × *dCK*/*RRM1* × *RRM2* expression ratio showed higher gemcitabine chemosensitivity, whereas cells with a lower ratio showed higher chemoresistance. The *hENT1* × *dCK*/*RRM1* × *RRM2* ratio significantly correlated with the gemcitabine IC_50_ in eight pancreatic cancer cell lines (*P*=0.0029). Neither the *RRM1* × *RRM2 ratio*, the *hENT1*/*RRM1* × *RRM2* ratio, nor the *dCK*/*RRM1* × *RRM2* ratio correlated with sensitivity to gemcitabine.

## DISCUSSION

Chemoresistance is a major cause of pancreatic adenocarcinoma treatment failure with gemcitabine. The majority of patients with gemcitabine-treated pancreatic adenocarcinoma become resistant after consecutive treatments, and fail to derive benefit from chemotherapy. Therefore, it is extremely important to clarify the mechanism behind chemoresistance and to identify predictive markers of inherent and acquired chemoresistance to gemcitabine for better treatment of these tumours.

It has been shown that modulation of cellular enzymes of gemcitabine transport and metabolism influences drug activity *in vitro* (Mackey *et al*, 1998, 1999; Goan *et al*, 1999; Ritzel *et al*, 2001). Cellular enzymes of gemcitabine transport and metabolism, that is, hENT1, dCK, RRM1, and RRM2, are well documented. Moreover, experimental data may improve the success of pancreatic cancer treatment either by selecting responsive patients or by modulation of gemcitabine effect with rationally selected drug combinations. For these reasons, this study addressed the transcription analysis of *hENT1*, *dCK*, *RRM1*, and *RRM2* in parental cell lines and in isolated variable resistant subclones continuously exposed to gemcitabine to determine possible predictive markers for gemcitabine resistance. Our data demonstrate for the first time that acquired and inherent chemoresistance of pancreatic cancer cells to gemcitabine is determined by the balance of *dCK, RRM1, RRM2*, and *hENT1* gene expression, but not to that of any of the individual genes. The *hENT1* × *dCK*/*RRM1* × *RRM2* expression ratio significantly correlates with resistance to gemcitabine in pancreatic cancer cells, including acquired gemcitabine-resistant cells, suggesting that a decrease of this ratio reflects inherent and acquired chemoresistance of pancreatic cancer cells to gemcitabine and may be a key to understanding the variable effectiveness of gemcitabine among individual patients. The expression ratio is a novel, informative marker for predicting and monitoring the responses of pancreatic cancer patients to gemcitabine.

A recent study performed on cultured cancer cell lines indicated that hENT1 is the major gemcitabine transporter in human pancreatic cancer cells (Garcia-Manteiga *et al*, 2003). It is speculated that populations of cells with lower hENT1 abundance may be relatively gemcitabine resistant owing to reduced intracellular accumulation. In fact, pharmacological inhibition of hENT1 in cells has been reported to render them gemcitabine resistant (Mackey *et al*, 1998). However, the current study shows that expression of the *hENT1* gene was not reduced in the development of gemcitabine resistance, and did not correlate with IC_50_ values of gemcitabine in eight pancreatic cancer cell lines. These data suggest that *hENT1* expression alone does not reflect inherent and acquired resistance to gemcitabine. The increase in *hENT1* expression in the PK1-G4000, PCI-G4000, and KLM1-G4000 subclones may be regarded as a compensatory adaptation to higher chemoresistance to gemcitabine.

Previous reports in cells and animal models cited *dCK* mutation/deficiency as the main mechanism for gemcitabine resistance in cells with an acquired resistance (Ruiz van Haperen *et al*, 1994; van der Wilt *et al*, 2000; Galmarini *et al*, 2001; Jordheim *et al*, 2003). In this study, expression of the *dCK* gene was slightly increased in subclones that were resistant to lower concentrations of gemcitabine, and was undetectable in subclones with high resistance to gemcitabine, that is, PCI43-G4000 and PK1-G4000. Furthermore, we found that expression of the *dCK* gene alone does not correlate with sensitivity to gemcitabine in eight pancreatic cancer cell lines. Deficiency of dCK described in previous reports was mainly based on studies performed on highly gemcitabine-resistant clones, and quite different from the clinical setting. Our data indicate that dCK deficiency is involved in a higher grade of acquired gemcitabine resistance, but not in a lower grade of resistant cells or in parental cells.

Expression of RR has been reported to be one of the determinants of gemcitabine chemoresistance in human tumour cells (Jung *et al*, 2001). In fact, artificial overexpression of RRM2 results in a further increase in gemcitabine chemoresistance (Goan *et al*, 1999). An increased level of RR expands the size of the dNTP pools, which competitively inhibits the incorporation of gemcitabine triphosphate into DNA (Plunkett *et al*, 1996). The expanded dNTP pools further downregulate the activity of dCK via a negative-feedback pathway. Results from this study demonstrate that *RRM1* mRNA expression was markedly increased in the KLM1 gemcitabine-resistant cells over that of the parental cells. This result supports the previous report that RRM1 is the marker predicting resistance to gemcitabine in lung cancer cell lines (Davidson *et al*, 2004). In this study, KLM1 gemcitabine-resistant cells had no *dCK* deficiency in contrast to the lack of *dCK* in PCI43 and PK1 gemcitabine-resistant cells. Therefore, these data demonstrate that RRM1 may correlate with acquired resistance to gemcitabine, especially in cells without dCK deficiency. *RRM2* mRNA was increased in PCI43 gemcitabine-resistant cells, but not in PK1 and KLM1 gemcitabine-resistant subclones. In the eight pancreatic carcinoma cells tested, the cells with higher inherent resistance to gemcitabine did not show higher levels of *RRM1* and *RRM2* expression. These results suggest that an increase in either *RRM1* or *RRM2* expression alone correlates with acquired chemoresistance, but does not reflect inherent chemoresistance to gemcitabine. In the development of gemcitabine resistance, increases in either *RRM1* or *RRM2* expression, in addition to a decrease in *dCK*, may indicate that cells are regulated to avoid damage by gemcitabine incorporation in gemcitabine-resistant cells.

Ribonucleotide reductase plays a role as a central enzyme controlling the rate of dNTP synthesis (Plunkett *et al*, 1996). It has recently been reported that the physiological factor for ribonucleotide reduction is thioredoxin, which is required for the RR reaction *in vitro* (Koc *et al*, 2006). In yeast, mutants lacking thioredoxin had significantly lower dNTP levels, supporting the idea that thioredoxin functions as an RR reductant *in vitro*. Interestingly, thioredoxin is identified as a gene whose basal expression is increased in pancreatic cancer cells in which Smad7 is commonly overexpressed (Arnold *et al*, 2004). Thioredoxin is downstream of smad7 in a pathway that acts to promote growth and induce apoptosis resistance in pancreatic cancer cells. Future studies are necessary to investigate whether or not thioredoxin is downregulated in gemcitabine-resistant pancreatic cancer cells.

Previous genomic analysis of pancreatic cancer has been performed exclusively with surgical and autopsy specimens, owing to the difficulty of tissue sampling without surgery. To overcome this difficulty, endoscopic ultrasonography-guided fine-needle aspiration biopsy (EUS-FNAB) is applied to obtain tumour cells as an effective and safe method for tissue diagnosis of pancreatic cancer (Agarwal *et al*, 2004). More recently, several investigators have demonstrated that genetic analysis using EUS-FNAB specimens is possible for determination of cancer stage or to improve the accuracy of diagnosis (Tada *et al*, 2002; Pellise *et al*, 2004). The current study shows that detection and quantitation of *hENT1*, *dCK*, *RRM1*, and *RRM2* mRNA can be performed rapidly and reliably using light cycler-PCR with SYBR Green fluorescence. The analytical strategy using light cycler-PCR with EUS-FNAB specimens will enable us to evaluate the chemoresistance of pancreatic cancer before and after gemcitabine treatment in the clinic.

In summary, quantitative RT–PCR analysis showed that the balance of four gene expression levels correlates with inherent and acquired resistance to gemcitabine in pancreatic cancer cells. We propose that resistance of cancer cells to gemcitabine is determined by the ratio of expression of these genes in pancreatic cancer cells. The ratio of gene expression may be a useful marker for predicting and monitoring the efficacy of gemcitabine therapy in pancreatic cancer patients. Further studies are currently underway using pancreatic cancer tissues obtained by EUS-FNAB to elucidate the importance of the expression ratio in gemcitabine therapy for pancreatic cancer patients.

## Figures and Tables

**Table 1:** Sequences of primers used in reverse transcription–PCR

**Gene**	**Forward primer (5′–3′)**	**Reverse primer (5′–3′)**
hENT1	AAAGGAGAGGAGCCAAGAGC	GGCCCAACCAGTCAAAGATA
dCK	CCCGCATCAAGAAAATCTCC	TCATCCAGTCATGCCAGTC
RRM1	GGAGGAATTGGTGTTGCTGT	GCTGCTCTTCCTTTCCTGTG
RRM2	CCCGCTGTTTCTATGGCTTC	CCCAGTCTGCCTTCTTCTTG
GAPDH	ATGACCACAGTCCATGCCAT	TTGAAGTCAGAGGAGACCAC

Abbreviations: dCK=deoxycytidine kinase; GAPDH=glyceraldehyde-3-phosphate dehydrogenase; hENT1=human equilibrative nucleoside transporter-1.

**Table 2:** IC_50_ values of gemcitabine in parental and gemcitabine-resistant cells of PCI43 and PK1 cell lines

**Cell lines**	**IC_50_ (nM) of GEM**	**Resistant ratio**
PCI43	350	
PCI43–G4000	55 000	157
PK1	160	
PK1–G4000	100 000	625
KLM1	80	
KLM1–G4000	210 000	2625

**Table 3:** Relative hENT1, dCK, RRM1, and RRM2 mRNA expression to GAPDH in human pancreatic cancer cell lines

		**Relative mRNA expression to GAPDH**			
**Cell lines**	**IC_50_ (nM)**	**hENT1**	**dCK**	**RRM1**	**RRM2**
PCI43	350	1.00±0.12	0.36±0.0.3	0.43±0.02	0.50±0.04
PK1	160	1.00±0.13	1.00±0.16	1.00±0.13	1.00±0.21
KLM1	80	3.88±0.22	0.81±0.02	1.08±0.04	1.46±0.13
PK9	70	1.12±0.17	0.99±0.05	0.31±0.02	0.63±0.06
PK8	40	3.45±0.28	2.51±0.07	0.71±0.04	1.82±0.19
MIAPaCa2	40	2.64±0.42	0.42±0.08	0.42±0.07	0.47±0.04
KPIN	40	23.26±1.33	2.59±0.04	1.58±0.02	4.31±0.41
BxPC3	30	3.52±0.51	1.20±0.21	0.70±0.08	0.84±0.16
*P*-value to IC_50_		NS	NS	NS	NS

NS: not significant.

Abbreviations: dCK=deoxycytidine kinase; GAPDH=glyceraldehyde-3-phosphate dehydrogenase; hENT1=human equilibrative nucleoside transporter-1.
